# Nephrogenic Systemic Fibrosis Risk and Liver Disease

**DOI:** 10.1155/2014/679605

**Published:** 2014-03-23

**Authors:** Robert F. Hanna, Lee A. Finkelstone, Daniel S. Chow, Vesselin Z. Miloushev, Mark R. Escudero, Stephen M. Lagana, Martin R. Prince

**Affiliations:** ^1^Department of Radiology, Columbia University, New York Presbyterian Hospital, 622 West 168th Street, PB-1-301, New York, NY 10032, USA; ^2^Division of Liver Diseases, Department of Pathology, Columbia University, 622 West 168th Street, New York, NY, USA

## Abstract

*Objective*. Evaluate the incidence of nephrogenic systemic fibrosis (NSF) in patients with liver disease in the peritransplant period. *Materials and Methods*. This IRB approved study retrospectively reviewed patients requiring transplantation for cirrhosis, hepatocellular carcinoma (HCC), or both from 2003 to 2013. Records were reviewed identifying those having gadolinium enhanced MRI within 1 year of posttransplantation to document degree of liver disease, renal disease, and evidence for NSF. *Results*. Gadolinium-enhanced MRI was performed on 312 of 837 patients, including 23 with severe renal failure (GFR < 30 mL/min/1.73 cm^2^) and 289 with GFR > 30. Two of 23 patients with renal failure developed NSF compared to zero NSF cases in 289 patients with GFR > 30 (0/289; *P* < 0.003). High dose gadodiamide was used in the two NSF cases. There was no increased incidence of NSF with severe liver disease (1/71) compared to nonsevere liver disease (1/241; *P* = 0.412). *Conclusion*. Renal disease is a risk factor for NSF, but in our small sample our evidence suggests liver disease is not an additional risk factor, especially if a low-risk gadolinium agent is used. Noting that not all patients received high-risk gadolinium, a larger study focusing on patients receiving high-risk gadolinium is needed to further evaluate NSF risk in liver disease in the peritransplant period.

## 1. Introduction 

Liver transplantation is the optimal treatment for patients with early-stage hepatocellular carcinoma (HCC) [1–3]. However, because the number of available livers is limited and does not meet demand, the United Network for Organ Sharing (UNOS) requires that transplant candidates with HCC be imaged preoperatively to select ideal candidates for liver transplantation. Current UNOS guidelines rely primarily on diagnostic imaging to preoperatively diagnose and stage tumors; preoperative biopsy is not required [4].

In a recent meta-analysis by Colli et al., gadolinium-enhanced MRI was suggested to be the superior imaging modality for diagnosing HCC [5]. However, there is a link between the administration of less stable gadolinium contrast agents (i.e., gadodiamide) and nephrogenic systemic fibrosis (NSF) in patients with severe renal failure (glomerular filtration rate (GFR) < 30 mL/min/cm^2^) [6–10]. Hence, potential hepatic transplant patients with severe renal insufficiency may have the benefits of gadolinium-enhanced MRI withheld due to concerns for developing NSF. Furthermore, a recently revoked Food and Drug Administration (FDA) advisory and mandated package insert labeling on all gadolinium agents extended the risk from severe renal disease alone to patients with even mild or moderate renal disease if there is coexistent liver disease. The warning is still active in Europe in accordance with the European Medicines Agency (EMA) guidelines [11]. The warning applies to patients in the perioperative liver transplant period and patients with hepatorenal syndrome. In the absence of data evaluating the association of liver disease with NSF while controlling for renal disease, the active EMA warning and historical FDA warning may result in less imaging, suboptimal imaging, or imaging with modalities with higher radiation exposure.

The recent meta-analysis by Mazhar et al., reviewing the NSF literature published between 2000 and 2008 found that patients with liver disease who develop NSF also have comorbid severe renal insufficiency but that liver disease alone does not confer a risk beyond that of the underlying renal insufficiency [12]. However, as the authors describe, the meta-analysis was limited by the summary nature of the reports, as many articles did not provide sufficient detail to consistently collect data regarding the nature of each patient's liver and kidney disease.

The purpose of our study was to evaluate the risk of NSF in hepatic transplant patients at a tertiary care liver transplantation center with 14 reported cases of NSF.

## 2. Materials and Methods

One author (MP) has patent agreements with GE Healthcare (Milwaukee, WI), Bracco Diagnostics (Princeton, NJ), Epix (Lexington, MA), Lantheus (N. Billerica, MA), and Bayer HealthCare Pharmaceuticals (Berlin, Germany). This retrospective, cross-sectional study was performed at a tertiary liver center and was HIPAA compliant. The investigational review board approved the study and waived patient consent requirements.

### 2.1. Subjects

Gadolinium-enhanced MR imaging is the routine clinical protocol for HCC surveillance at our institution. We analyzed records of all patients who underwent liver transplantation from January 2003 to January 2013. Although Gadolinium-enhanced MRI entered medical practice in 1988, NSF was not diagnosed at our institution prior to 2003; hence, January 2003 was selected as our start date for review.

### 2.2. Renal Status

Electronic medical records were analyzed for the number and dates of MRI exams before and after liver transplant. Patients were excluded if they did not have a gadolinium-enhanced MRI performed at our institution within 1 year posttransplantation, as it was our aim to analyze the risk of NSF in liver disease in the peritransplant period. We also did not analyze patients with hepatorenal syndrome because their life expectancy, which varies from 2 weeks to 6 months depending on if they have type 1 or type 2 disease, is short and thus often those patients do not live long enough to develop NSF. The estimated glomerular filtration rate (eGFR) of the included patients was obtained from lab results of the most recent pre- and/or posttransplant MRI (within one year of MRI examination date). As awareness of the link between NSF and less stable gadolinium contrast agents (i.e., gadodiamide) began to spread in 2006, the practice of obtaining creatinine values was obtained within 2 weeks of gadolinium administration, as is the current policy at our institution [7, 8]. The eGFR was calculated by using the Modification of Diet in Renal Disease (MDRD) equation (http://nephron.com/cgi-bin/MDRDSIdefault.cgi). In order to calculate a Child-Pughes score prior to transplant, the bilirubin, albumin, International Normalized Ratio (INR), and degree of ascites and encephalopathy were recorded. In order to calculate a Model for End Stage Liver Disease (MELD) score prior to transplant, the bilirubin, INR, and creatinine were recorded. For patients who had been regularly dialyzed, a creatinine of 4 was used according to UNOS guidelines [4].

### 2.3. Determination of NSF

Dermatology and dermatopathology records were reviewed to locate patients with biopsy confirmed NSF. For each NSF case, the dermatology consultation notes were reviewed to determine the onset and severity of symptoms. Records were then further reviewed to determine if each patient had any history of liver disease and if they were in the peritransplant period. The Child-Pughes, MELD score, creatinine, and GFR of all NSF patients were recorded.

### 2.4. Gadolinium

The administration of gadolinium to these patients was confirmed by reviewing MR imaging records. The gadolinium agents administered prior to June 2007 were gadopentetic acid, gadobenate, and gadodiamide. Subsequently thereafter, only gadobenate, gadoteridol, gadoxetate, and gadofosveset were administered at our institution. Standard dose administrations were based on patient weight (0.1 mmol per kilogram of body weight). High doses of gadolinium were administered as fixed volumes of 20, 30, or 40 mL for liver MR imaging, which corresponded to approximately 0.2–0.4 mmol/kg. High dose administration was used routinely from liver MRI prior to July 2006, and weight-based dosing (0.1 mmol/kg) was utilized afterwards.

### 2.5. Statistical Analyses

Fisher's exact test was used to examine whether there were any significant differences in the presence of NSF in patients with severe chronic kidney disease (defined as eGFR < 30 mL/min/cm^2^) and in patients with severe liver disease (defined as Child-Pugh Class C). Additionally, a subanalysis of patients with severe kidney disease was performed to assess for any associations with severe liver disease in this population using Fisher's exact test.

All *P*-values were two-tailed, and a *P*-value < 0.05 was considered significant. Statistical power was also calculated. Analysis was performed using Statistical Package for Social Sciences (SPSS) version 20.0 for Microsoft Windows (SPSS Inc., IL, USA). The* R* statistical program was also used to compute the *P*-values using Fisher's exact test for Table 4 and comparing the observed rate of NSF with the published institutional rate as stated in the discussion [13]. The* R* statistical program was also used to compute the upper limit of a Bayesian credible interval from a binomial distribution based on the observed rate using the binomial package for* R* [14].

## 3. Results

### 3.1. Subjects

**Figure 1:**
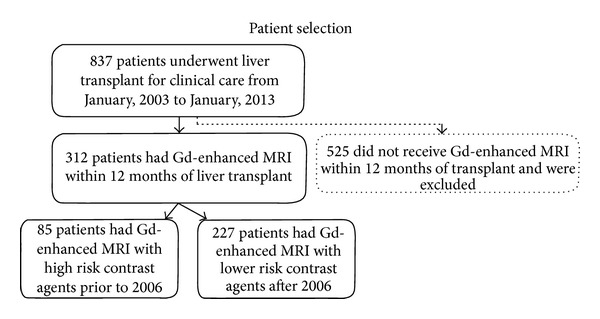
Flow chart of patient selection. Solid lines specify patients who were included in the study and dotted line specifies patients who were excluded.

From January 2003 to January 2013, our institution performed gadolinium-enhanced MRI exams on 837 transplantation patients. Of these 837 patients (Figure 1), 525 did not receive gadolinium-enhanced MRI within 12 months of transplant and were excluded. Of the remaining 312 patients (204 men, 108 women; mean age 54 years, range 1–74: see Table 1), 237 (75.9%) had gadolinium-enhanced MRI both before and after transplantation, 53 patients (16.9%) had an MRI only prior to transplantation and 22 only after transplant (7.1%). A total of 1,742 gadolinium-enhanced MRI examinations were performed on these 312 patients; 120 patients (38.5%) had 1–3 MRI exams, 91 patients (29.2%) had 4–6 exams, and 101 patients (32.3%) had 7 or more exams. Of the 312 patients, 85 underwent MRI with high-risk contrast agents prior to 2006 and 227 underwent MRI with more stable contrast agents after 2006.

Patients with liver disease included 164 (52.6%) with cirrhosis but without HCC, 124 (39.7%) with cirrhosis and HCC, and 24 (7.7%) with HCC but without cirrhosis. The median MR imaging to transplant time interval was 109 days (range of 1–347 days). Child-Pughes scores of the patients included 142 class A (46%), 99 class B (32%), and 71 class C (23%). The range of eGFR observed is listed in Table 1. The associated average MELD score was included in Table 1. Of the 312 patients, 105 (33.7%) had no renal failure (GFR > 90), 109 (34.9%) had mild renal failure (GFR 60–90), 75 (24.0%) had moderate renal failure (GFR 30–59), and 23 (7.3%) had severe renal failure (GFR < 30 cc/min/1.73^2^). Patients with severe renal disease had an increased risk for NSF and patients with severe liver disease did not (Table 2).

The two patients with liver disease who experienced NSF both received high doses of gadodiamide. During their course of multiple MRIs at our institution, patient 1 received a single high dose (>40 mL) and patient 2 received three high doses of gadodiamide within a one-month span. All other doses were smaller doses and utilized gadopentetic acid. In total, patient 1 had 1 pretransplant MRI (4 days before surgery) and 2 posttransplant MRI (shortest interval 13 days after transplant); patient 2 had 1 pretransplant MRI (12 days before surgery) and 4 posttransplant (shortest interval 358 days after transplant). Their respective Child-Pughes scores were 12 and 9, MELD score 42 and 36, and each had serum creatinine and eGFR of 6.4 mg/dL, 13.7 mL/min/1.73 m^2^ (dialysis dependent) and 8.3 mg/dL, 14.7 mL/min/1.73 m^2^.

### 3.2. Analysis of Risk Factors

Of the 14 cases of NSF at our institution, 13 had chronic renal disease; one had acute renal disease due to acute pancreatitis. Only two of the fourteen had liver disease. Our study had a standard deviation of 0.079, which yielded a power of 0.71. There was a significant association between severe renal disease (GFR < 30* *mL/min/1.73 m^2^) and NSF (*P* = 0.03). The upper limit of a 95% credible interval for the incidence of NSF in patients with mild and moderate renal failure was 1.8%. However, no association between severe liver disease and NSF was present (*P* = 0.41) (Table 2). In patients with severe renal disease, there is no significant association between NSF and severe liver disease (*P* = 0.998) (Table 3).

## 4. Discussion

Diagnostic imaging plays a critical role in pretransplantation staging for HCC. Specifically, transplantation may be expedited in patients with stage T2 tumors based on MR findings without requiring biopsy. Patients whose tumor burden on MR exceeds transplantation guidelines will be delisted [15, 16]. This critical role for gadolinium-enhanced MRI may be obviated by active EMA and historical FDA warnings of increased NSF risk in patients with liver disease with kidney disease of any severity [12, 17, 18].

At least three major academic centers report screening for liver disease in patients who will undergo gadolinium-enhanced MRI [9, 10]. They cite the historical FDA label and articles describing an association between liver disease and NSF [19–21]. One study is a case-report describing a hepatitis C liver transplant patient who developed cyclosporine induced renal failure necessitating dialysis 11 years after transplant; a second study describes a patient with alpha-1 antitrypsin related cirrhosis and end stage renal disease from membranous nephropathy; and a third study describes a patient with hepatitis B and C cirrhosis status after failed transplant for portal vein thrombosis with end stage renal disease requiring hemodialysis [19–21]. All 3 patients had severe, dialysis-requiring end stage renal disease. These case reports have identified that NSF occurs in patients with liver disease but there does not appear to be any published data establishing liver disease as an independent risk factor.

The suggested association between NSF and liver disease has legal ramifications as well. Plaintiffs for anti-gadolinium lawsuits are actively solicited based on a history of prior liver disease [22]. These developments suggest that a liver disease-NSF association has gained some acceptance in the absence of well-controlled, scientifically valid data. The threat of litigation may lead physicians to avoid gadolinium-enhanced MRI resulting in suboptimal care for a patient population in which such imaging plays a critical role.

A recent meta-analysis by Mazhar et al. showed that amongst 112 articles, only 335 unique NSF cases were described, of which 41 patients had liver disease. Of these 41, only one did not have severe renal insufficiency (GFR = 34) [23]. Additionally, authors noted that this patient received nearly 750% of the recommended gadolinium dose in a short time interval. Within our study, we examined 312 patients with liver disease in the peritransplant period, of which two developed NSF. These two patients received larger than normal doses of nonionic linear gadolinium and were also in severe renal failure. However, no cases of NSF were identified in periliver transplantation patients without severe renal disease. Furthermore, no statistically significant association was present between severe and nonsevere liver disease for the development of NSF. Additionally, a subanalysis of patients with severe renal disease revealed that severe liver disease had no significant association with the incidence of NSF.

A limitation of our study was that determination of eGFR in patients with cirrhosis is imperfect and may be overestimated [24]. Calculation of eGFR was based on serum creatinine, which may be reduced with impaired hepatic metabolism of creatine to creatinine in hepatic insufficiency. This may result in a higher apparent rate of NSF in cirrhotic patients with renal insufficiency classified as nonsevere.

A second limitation given our retrospective study design was the possible underestimation of the number NSF cases. We carefully reviewed dermatology/dermatopathology records, and patients were considered to have NSF only if biopsy verification was demonstrated. Although to our knowledge, there were no patients who were suspected of having NSF based on the dermatology records that did not undergo skin biopsy. There is an extremely small possibility that mild cases of NSF may not have been observed by patients or not believed to be severe enough to warrant referral to dermatology. If either was the case, then the actual incidence of NSF may have been higher than that reported. However, skin disease that is not severe enough or that does not persist long enough to justify biopsy is not likely to be clinically significant. Furthermore, patients may have had dermatology consults outside of our institution, which would also lead to an underestimation of the number of cases of NSF, but our transplant patients are followed extremely closely inside our own institution. Regarding this, there is no evidence of referrals from outside dermatology centers for further work-up.

A third limitation of our study was the small number of patients in our study sample. Only 312 out of our 837 liver transplant patients were included in the final study population to meet our aim of analyzing NSF in the peritransplant period, which we defined to be within 1 year of transplant. This small patient size limited the power of our study, which was 0.71. Although our power was still relatively high, due to the rare incidence of NSF in the peritransplant period in our population, an ideal power of 0.8 or higher was not achieved. However, our institution performs a high number of gadolinium-enhanced MRIs and has had 14 total cases of NSF, which is not an insignificant number if the total number of reported cases worldwide is considered. Therefore, our data can be considered a strong foundation for future studies and will likely mirror results of future, larger studies. Thus, future studies comprised of multicenter, larger patient populations would be of benefit to increase the power of the study and corroborate or disprove our conclusions regarding the risk of NSF in the peritransplant period, especially in patients that received high-risk gadolinium contrast agents.

Lastly, we did not do an analysis of patients with hepatorenal syndrome, as is described in the second part of the FDA liver disease-related gadolinium warning. This is because depending on whether a patient has type I or type II hepatorenal disease, their life expectancy is only 2 weeks to 6 months [25]. Thus, many patients do not often live long enough to get NSF. We made a conservative decision to exclude these patients even though excluding them decreased our total number of patients and the power of the study.

However, when comparing our proportion of NSF in severe renal failure (2/23) within our institutional NSF database (12/501, as published in Prince et al., [26]), no statistical significance was observed after accounting for overlap between populations (*P* = 0.10). Furthermore, all of the patients who incurred NSF before 2006 received high dose linear nonionic gadolinium, which has since been replaced with ionic and macrocyclic agents [27]. Since 2006, patients with GFR < 30 mL/min/1.73 m^2^ either do not receive gadolinium or are dialyzed within 24 hours. There are no such precautions in liver disease patients.

## 5. Advances in Knowledge

Data at our institution suggests that liver disease confers little to no association with nephrogenic systemic fibrosis (NSF) beyond what is expected based upon the underlying renal function. There have been no cases of NSF since 2006 in spite of a third of our liver disease patients receiving 7 or more gadolinium-enhanced MRI examinations in the peritransplant period. Our two cases of NSF were in patients who received high doses of less stable gadodiamide before 2006 and no cases of NSF were identified after 2006 once our institution switched to more stable gadolinium contrast agents.

## 6. Implications for Patient Care

Gadolinium insert warnings, the threat of litigation, and recent contemporaneous studies may inappropriately deter physicians from requesting contrast-enhanced MRI examinations in patients with liver disease. This deterrent may be exaggerated by the fact that traditionally the risk of NSF has been associated with less stable gadolinium contrast agents (i.e., gadodiamide), which are currently rarely used in clinical practice in favor of newer, more stable contrast agents. This study supports the safety of gadolinium-enhanced MRI in patients with liver disease in the peritransplant period.

## 7. Conclusion

In summary, we found in our small sample size that liver disease confers little to no association with NSF beyond what is expected based upon the underlying renal function in spite of a third of our liver disease patients receiving 7 or more gadolinium-enhanced MRI examinations. The historical FDA gadolinium related warning, the active EMA warning, threat of litigation, and recent contemporaneous studies may inappropriately deter physicians from requesting contrast-enhanced MRI examinations in patients with liver disease. This study supports the safety of gadolinium-enhanced MRI in patients with liver disease in the peritransplant period, especially if a low-risk gadolinium agent is used. Noting that not all patients received high-risk gadolinium contrast agents, a larger study focusing on patients receiving only high-risk/less stable gadolinium is needed to further evaluate the risk of NSF in liver disease in the peritransplant period.

## Figures and Tables

**Table 1:** Demographics of patients who had undergone liver transplantation with documented GD exposure. Numbers in parenthesis represent percentages. MELD represents the Model for End Stage Liver disease score average. GFR units equal mL/min/1.73 m^2^.

Demographics of transplant patients with gadolinium-MRI (*N* = 312)		
Male/female	204/108	
Mean age at transplant, yr (±SD)	54.7 (±12)	
Mean/median Gd pretransplant time	81/109 days	
Mean/median Gd posttransplant time	139/208 days	
Mean/median doses of Gd	5.3/5 doses	

Number of exams per patient		
1–3	120 (38.4)	
4–6	91 (29.2)	
7 or more	101 (32.4)	

Cirrhosis by Child-Pughes score		MELD
Class A	142 (45.5)	11.7
Class B	99 (31.7)	22.5
Class C	71 (22.8)	33.7

Renal disease		
GFR 0–30	23 (7.4)	
GFR 30–59	75 (24.0)	
GFR 60–89	109 (34.9)	
GFR > 90	105 (33.7)	

**Table 2:** NSF and severe renal and severe liver disease. Severe renal disease was found to have a significant association with NSF compared to nonsevere renal disease. Severe liver disease did not have a significant association with NSF compared to nonsevere liver disease. MELD represents the Model for End Stage Liver disease score average.

Characteristics	NSF negative	NSF positive	*P* value
Renal disease			0.03
GFR > 30	289	0	
GFR 0–29	21	2	
Liver disease			0.412
Class A and B (mild/mod.) (MELD avg. 11.7 and 22.5)	241	1	
Class C (severe) (MELD avg. 33.7)	69	1	

**Table 3:** In patients with severe renal disease, severe liver disease did not have an association in increasing the risk of NSF. MELD represents the Model for End Stage Liver disease score average.

Characteristics	NSF negative	NSF positive	*P* value
Severe renal disease			0.998
Class A and B (MELD avg. 11.7 and 22.5)	13	1	
Class C (MELD avg. 33.7)	8	1	

**Table 4:** Table 4 demonstrates the association of NSF in patients with liver disease and varying degrees of renal failure. The mild to moderate renal group that the Gd warning label targets had 0 cases of NSF.

Characteristics	Patients	NSF cases
Liver + no renal disease	183 (58.8%)	0 (0%)
Liver + mild/mod. renal (GFR 30–90) Dz	106 (34.3%)	0 (0%)
Liver + severe renal Dz (GFR < 30)	21 (6.9%)	2 (100%)

Total	310	2
